# Autism symptoms, functional impairments, and gaze fixation measured using an eye-tracker in 6-year-old children

**DOI:** 10.3389/fpsyt.2023.1250763

**Published:** 2023-10-02

**Authors:** Toko Mori, Kenji J. Tsuchiya, Taeko Harada, Chikako Nakayasu, Akemi Okumura, Tomoko Nishimura, Taiichi Katayama, Masayuki Endo

**Affiliations:** ^1^Faculty of Nursing, Shijonawate Gakuen University, Osaka, Japan; ^2^Research Center for Child Mental Development, Hamamatsu University School of Medicine, Hamamatsu, Japan; ^3^Department of Child Development, United Graduate School of Child Development, Osaka University, Kanazawa University, Hamamatsu University School of Medicine, Chiba University, and University of Fukui, Osaka, Japan; ^4^Division of Health Sciences, Osaka University Graduate School of Medicine, Osaka, Japan

**Keywords:** autism spectrum disorder, functional impairment, gaze, Gazefinder, preschooler, general population

## Abstract

**Introduction:**

Autism spectrum disorder (ASD) is a neurodevelopmental disorder clinically characterized by abnormalities in eye contact during social exchanges. We aimed to clarify whether the amount of gaze fixation, measured at the age of 6 years using Gazefinder, which is an established eye-tracking device, is associated with ASD symptoms and functioning.

**Methods:**

The current study included 742 participants from the Hamamatsu Birth Cohort Study. Autistic symptoms were evaluated according to the Autism Diagnostic Observation Schedule, Second Edition (ADOS-2), and the functioning of the participating children in real life was assessed using the Japanese version of the Vineland Adaptive Behavior Scales, Second Edition (VABS-II). The Gazefinder system was used for gaze fixation rates; two areas of interest (eyes and mouth) were defined in a talking movie clip, and eye gaze positions were calculated through corneal reflection techniques.

**Results:**

The participants had an average age of 6.06 ± 0.14 years (males: 384; 52%). According to ADOS, 617 (83%) children were assessed as having none/mild ASD and 51 (7%) as severe. The average VABS-II scores were approximately 100 (standard deviation = 12). A higher gaze fixation rate on the eyes was associated with a significantly lower likelihood of the child being assigned to the severe ADOS group after controlling for covariates (odds ratio [OR], 0.02; 95% confidence interval [CI], 0.002–0.38). The gaze fixation rate on the mouth was not associated with ASD symptoms. A higher gaze fixation rate on the mouth was associated with a significantly lower likelihood of the child being assigned to the low score group in VABS-II socialization after controlling for covariates (OR, 0.18; 95% CI, 0.04–0.85). The gaze fixation rate on the eyes was not associated with functioning.

**Conclusion:**

We found that children with low gaze fixation rates on the eyes were likely to have more ASD symptoms, and children with low gaze fixation rates on the mouth were likely to demonstrate poorer functioning in socialization. Hence, preschool children could be independently assessed in the general population for clinically relevant endophenotypes predictive of ASD symptoms and functional impairments.

## 1. Introduction

Autism spectrum disorder (ASD) is a neurodevelopmental disorder characterized by deficits in social interaction and communication, as well as restricted interests and repetitive behaviors (1).

Clinically, ASD is characterized by abnormalities in eye contact during social exchanges with others (1). Experiments have led to the successful identification of atypicalities in orienting to eye gaze cues of a human figure on screen (2–5). These findings have also been explained in the context of social attention (6). Despite the inherent ambiguity of the concept of social attention, multiple studies have focused on atypicalities in social attention in children with ASD while also measuring the amount of their gaze fixation on the human face as well as the eye and mouth regions on a human face presented on a screen (7, 8). The amount of gaze fixation has been associated with the observed degree of atypicalities in social interactions and communication (9–11). The characteristics of atypical social attention have also been reported in very young children without a definite diagnosis of ASD but instead at high familial risk for ASD who have not yet started speaking. For instance, infants at high familial risk for ASD have been observed to show differences in visual attention to social stimuli compared with low-risk infants as early as 1 week after birth, and these abnormalities persist at 4 months of age (12, 13). In a study involving infants diagnosed with ASD at 6 months of age, a decrease in gaze toward people and faces within social scenes has been reported (14). Additionally, a reduced gaze toward shared activities with others has been observed in children with ASD at 20 months of age (15). Furthermore, developmental changes in gaze fixation have been reported, indicating that gazing toward faces and eyes decreases with age in individuals with ASD [6 months to 3 years (16); 3 to 17 years (17)]. These findings suggest that the atypical features of social attention, reflected in gaze, in individuals with a diagnosis of or at risk of ASD are evident from an early age and may serve as potential early biomarkers for the ASD phenotype. However, some studies have not reported any differences between individuals with and without ASD regarding the amount of gaze fixation on specific areas of interest (AOIs) (18, 19). A small number of studies have also not observed any associations between the amount of gaze fixation and the degree of atypicalities in social interactions and communication (20).

The inconsistencies in the aforementioned findings have led to two unanswered questions: First, most of the studies conducted thus far have compared individuals with and without ASD. This type of comparison could have introduced potential biases into the data as children with ASD were assessed to formulate reliable diagnoses. In contrast, the included children without ASD were treated as not having a diagnosis of ASD, while they may have subthreshold autistic symptoms. If children with higher but subthreshold levels of autistic symptoms are enrolled, the comparison between children with and without ASD may produce only a small difference. However, a larger difference may be observed between the two groups if children with a minimal level of autistic symptoms were included in the non-ASD group. These considerations suggest that the inconsistency in previous studies was due to the variability of the comparison group. Second, while studies have reported the association of social attention measures with the degree of atypicalities observed in social interaction and communication (21, 22), the association of social attention measures with difficulties in functioning has been tested in some studies (23, 24). This is an important perspective since “clinically significant impairment in social, occupational, or other important areas of current functioning” (The Diagnostic and Statistical Manual of Mental Disorders, Fifth Edition; DSM-5) (1) is the necessary criterion to formulate the diagnosis of ASD. Functioning refers to the ability to engage in independent actions and practical activities in daily life rather than the capacity to acquire skills that involve intellectual abilities (25). Functioning has been addressed in individuals with ASD and has been shown to be associated with ASD symptoms. For instance, the increased severity of autism was associated with lower functioning in socialization, communication, and daily living skills (26, 27). However, a factor analysis of the phenotypic variation in ASD introduced two distinct components, namely autistic symptoms and level of functioning (28), which were also suggested to be considered when diagnosing ASD in DSM-5. Functioning also matters in clinical courses. For example, it is possible for some children with more severe and stable autistic symptoms to show significant improvement in adaptive functioning, whereas some children with mild autistic symptoms show lower and worsening adaptive functioning (29). Intriguingly, infants who later had ASD symptoms and functional impairment had a pattern of gaze fixation on human faces distinct from the pattern shown by infants with the symptoms but without functional impairment (30).

To address the above two questions, the association between social attention and each ASD symptom or functional impairment, rather than individuals with and without an ASD diagnosis, would be a new approach to resolving the inconsistencies in the results of previous studies. We aimed to clarify whether the amount of gaze fixation measured at the age of 6 years using Gazefinder, which is an established eye-tracking device, is associated with ASD symptoms and functioning.

We hypothesized that gaze fixation rates on the human face could serve as indicators reflecting ASD symptoms and functioning separately. Furthermore, we focused on potential linear or non-linear relationships between gaze fixation rates and ASD symptoms or functioning.

## 2. Methods

### 2.1. Participants

This study was conducted as part of an ongoing prospective cohort study, the Hamamatsu Birth Cohort Study for Mothers and Children (HBC Study), which includes mothers (*n* = 1,138) and their children (*n* = 1,258) (31, 32). Initially, all women in their first or second trimester of pregnancy who visited the Hospital of Hamamatsu University School of Medicine or the Kato Maternity Clinic between November 2007 and March 2011 were invited. Informed consent was obtained from participants in this study. The two research sites are both located in the northeastern part of Hamamatsu City, a city with a population of approximately 800,000 in the central part of Japan. The participating women at the time of enrollment were a fairly representative sample of pregnant women from the community and Japan (31, 32). Even though the university hospital provides specialized maternity services, the children who were born to these pregnant women whose gestational age was <37 weeks were 7% and those with a birth weight of <2,500 g were 13%, indicating that these figures did not show a significant departure from the national statistics (5 and 10%, respectively).

The current study included 761 participants among those enrolled in the HBC Study who had also completed the assessments, including eye gaze measurement using Gazefinder, at approximately 6 years of age.

### 2.2. Clinical measures

#### 2.2.1. Autistic symptoms

In this study, we defined the standardized scores of the Autism Diagnostic Observation Schedule, Second Edition (ADOS-2) as representative of autistic symptoms. ADOS is the specific gold-standard diagnostic test for ASD. It is a standardized, semi-structured assessment of items evaluating social affect (SA) domain and restricted interests, and repetitive behaviors (RRB) domain of ASD (33). Calibrated severity scores (CSS) are used to equalize total scores across all modules of ADOS (34). CSS total scores range from 1 to 10. Subjects are classified according to CSS as follows: “none” (1–2), “mild” (3–4), “moderate” (5–7), or “severe” (8–10). To detect any clinically meaningful association, we collapsed “none” and “mild” categories since most children in “mild” categories are not considered to have ASD, both in the Japanese (35) as well as in the original version of the ADOS (36). In this study, module 3 of ADOS-2 was equally administered to all the participating children. In module 3, target individuals who have developed the ability to speak fluently (equivalent to or higher than the expressive language skills of a typically developing 4-year-old) and are of an appropriate age to engage with toy figures based on the content of the test.

#### 2.2.2. Functioning

In this study, we defined the standardized scores of the Japanese version (25) of the Vineland Adaptive Behavior Scales, Second Edition (VABS-II) (37) as representative of functioning. VABS-II assesses adaptive behavior through a semi-structured interview with a person familiar with the subject's daily life (usually the mother of the participating child), with standardized scores for three domains (socialization, communication, and daily living skills) and a mean standard score of 100 and a standard deviation (SD) of 15. Higher scores indicated better functioning. Following the previous studies (25), we classified the VABS-II score into three groups: low = average – 1 SD (standardized score ≤ 85); average (standardized score 86–114); and high = average + 1 SD (standardized score ≥ 115). The motor skills score was not used in this study because the motor skills domain was applicable only to young children up to approximately 6 years of age.

#### 2.2.3. Measurement of eye gaze

We used Gazefinder in this study. It is a system used to capture the sequence of eye gaze that is evoked by movie clips. The system is equipped with a 19-inch monitor (1,280 × 1,024 pixels), manufactured by JVCKENWOOD Corporation. (Yokohama, Japan). Corneal reflection techniques enable the device to calculate eye gaze positions on the monitor as (X, Y) coordinates in pixel units at a frequency of 50 Hz. We used the eye gaze data elicited by a human face in a movie clip. In the selected movie clip, a woman talks with her eyes fixed and looking ahead. The details of this device as well as the stimuli have been described previously (17, 38).

We defined the two AOIs (eyes and mouth) in squares on the talking movie clip (7 s). The areas were specified with x and y axes on the monitor. The gaze fixation rate was defined as the proportion of gaze fixation time allocated to each AOI (eyes, mouth, and overall) divided by the duration of each movie clip. The gaze fixation rate was between 0.0 and 1.0; it represents the focus on the object (i.e., the higher the gaze fixation rate, the more intensively the child focused on the object). The sum of gaze fixation rates on the eyes and mouth is not equal to the overall gaze fixation rate for the talking moving clip because gaze fixation rates other than eyes and mouth were also measured. However, this measure is outside the scope of our interest.

**Figure 1:**
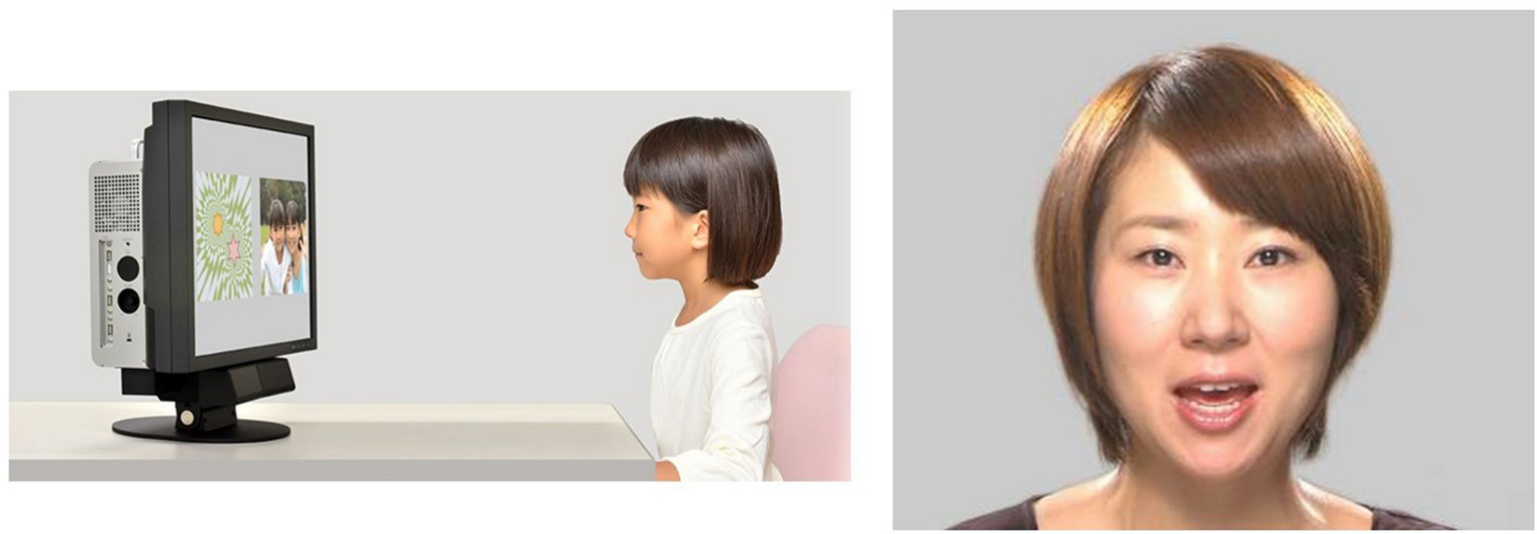
**(Left)** Experimental setup. The camera used to evaluate eye gaze patterns is placed under the screen, and the child can focus on the screen. **(Right)** Movie clip used in the study. A woman talks with her eyes fixed and looking ahead. We had permission to use the Gazefinder samples from JVCKENWOOD Corporation.

The child was asked to sit on a chair in front of the screen alone without any restrains or equipment placed on the child's body (Figure 1). Since Gazefinder does not require the device to be attached to the child, the burden on the child is minimized (17, 38–40).

### 2.3. Statistical analysis

First, we calculated attributes (age and sex), ASD symptoms, functioning, and the overall gaze fixation rate. Next, we analyzed the relationship between the gaze fixation rates for the two AOIs (eyes and mouth) and ASD symptoms. The ADOS CSS, which reflects the level of ASD symptoms, was not normally distributed. To avoid using linear regression with the intention of not compromising interpretability, we classified the ADOS CSS into three groups as the dependent variable (none/mild = 1–4; moderate = 5–7; severe = 8–10) and performed multinomial logistic regression analysis instead. We entered the gaze fixation rate on the eyes and on the mouth, and the overall gaze fixation rate as independent variables. Following the previous studies (26, 41, 42), the sex of the child and the mother's age and education in years were used as covariates in relation to autistic traits and functioning. Correlation analyses were performed between each gaze fixation rate (eyes, mouth, and overall) and the ADOS CSS (model 1). A multinomial logistic regression analysis was also performed between all gaze fixation rates (eyes, mouth, and overall) and the ADOS CSS (model 2); odds ratios and 95% confidence intervals (95% CI) were also calculated. If we are to find any association between ADOS CSS and the gaze fixation rates, we repeated the analysis while replacing the three groups based on ADOS CSS with the tertile of either the SA or RRB domain score of ADOS-2.

Finally, we analyzed the relationship between the gaze fixation rate for the two AOIs and their functioning. We performed multinomial logistic regression analysis for comparable interpretability using the three groups (low, average, and high) of the VABS-II scores. We also used the gaze fixation rate on the eyes, on the mouth, and the overall gaze fixation rate as independent variables and the sex of the child, the mother's age, and education in years as covariates. Correlation analyses were performed between each gaze fixation rate (eyes, mouth, and overall) and VABS-II score classification (model 1). Multinomial logistic regression analysis was performed between all gaze fixation rates (eyes, mouth, and overall) and the VABS-II score classification (model 2); odds ratios and 95% confidence intervals were also calculated.

If any of the associations were shown to be significant, we iterated the analyses using ordered logistic regression to test the linearity.

These analyses were conducted using Stata 15.0.

### 2.4. Ethical statement

This study was approved by the institutional review board of the Hamamatsu University School of Medicine. Written informed consent was obtained from all caregivers for their participation as well as for their child's participation in the study.

## 3. Results

### 3.1. Participant attributes

Following the protocol described in our previous study using Gazefinder (38), we excluded 19 children from the analysis who had a gaze fixation rate of < 50% throughout the measurement using Gazefinder. Ultimately, 742 patients were analyzed. The participants had an average age of 6.06 ± 0.14 years, and 384 (52%) of them were male children. According to ADOS CSS, 617 (83%) children were assessed as having none/mild ASD. In contrast, 51 (7%) children were assigned to the severe group with an ADOS CSS of 8 to 10. As expected, the average VABS-II scores for the three domains were approximately 100, and the SD was approximately 12 (Table 1).

**Table 1:** Characteristics of the participants.

	**N**	**Means**	**SD**	**Range**
Age (years)	742	6.06	0.14	5.57–6.97
ADOS score	740	2.44	2.33	1–10
ADOS severity	740			
None/mild	617	1.51	0.89	1–4
Moderate	72	5.96	0.78	5–7
Severe	51	8.76	0.79	8–10
VABS-II	742			
Communication		100.51	12.57	67–135
Daily living skills		98.82	11.16	54–124
Socialization		98.80	12.03	53–127
Overall gaze fixation rate	742	0.88	0.17	0–1.00
Gaze fixation rate on the eyes	742	0.24	0.16	0–0.96
Gaze fixation rate on the mouth	742	0.51	0.21	0–0.97

ADOS, Autism Diagnostic Observation Schedule; VABS-II, Vineland Adaptive Behavior Scales, Second Edition.

### 3.2. Relationship between gaze fixation rate and ASD symptoms

**Figure 2:**
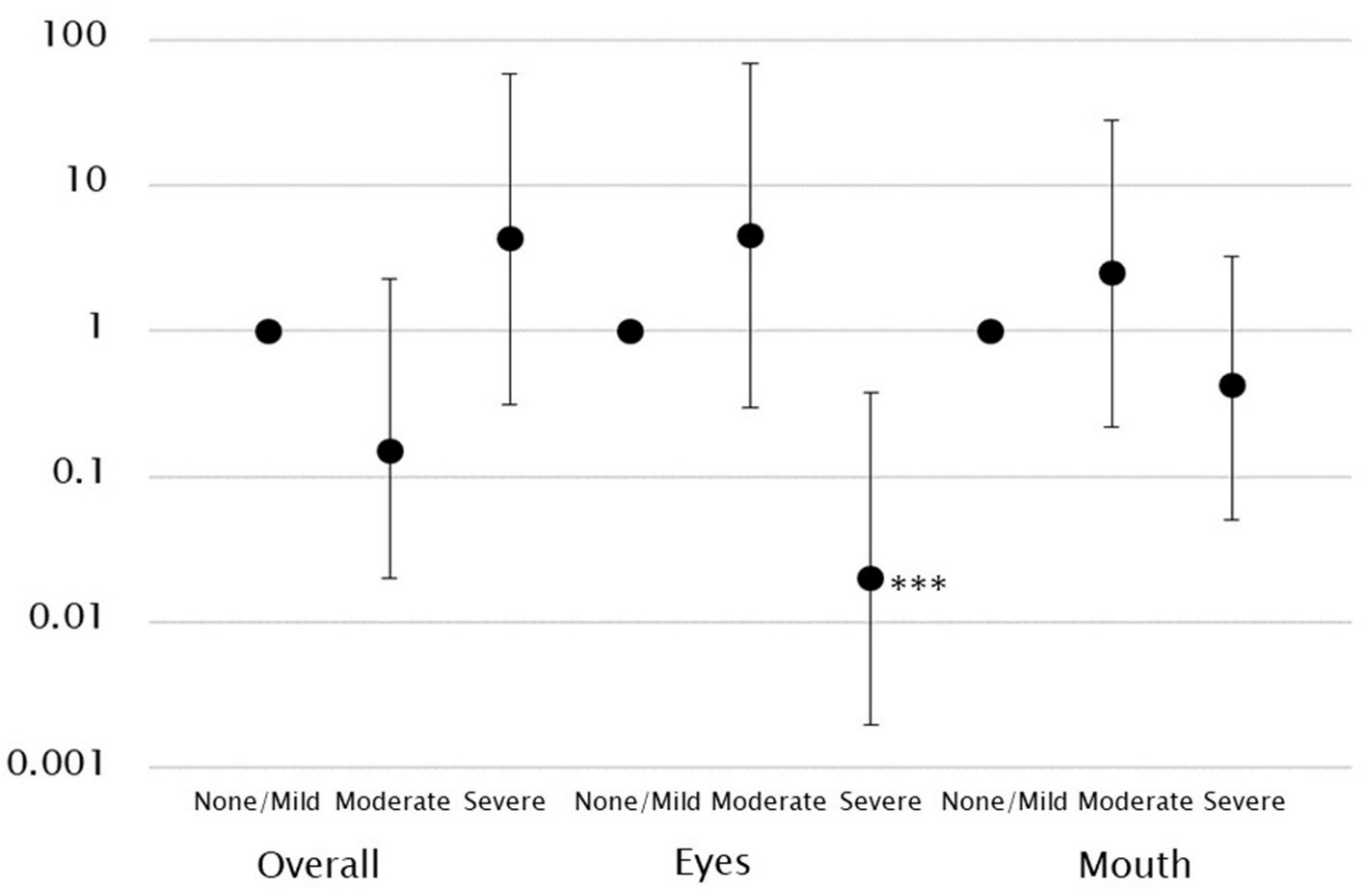
Log-scale representation of odds ratios and 95% confidence intervals indicating the association between gaze fixation rates and ASD symptoms classification. There was an association between low gaze fixation rates on the eyes and severe ASD symptoms. ***p < 0.001.

A higher gaze fixation rate on the eyes was associated with a significantly lower likelihood of the child being assigned to the severe ADOS CSS group. This was also observed after controlling for covariates (model 2: OR, 0.02; 95% CI, 0.002–0.38). Hence, this indicates that the lower the gaze fixation rate on the eyes, the more likely the child is to have severe symptoms. However, no such association was observed in the other groups. The gaze fixation rate on the mouth was not associated with ASD symptoms (Table 2, Figure 2).

**Table 2:** Relationship between the severity of ASD symptoms and gaze fixation rate (*n* = 740).

		**n**	**Model 1 Odds ratio (95% CI)**	**Model 2 Odds ratio (95% CI)**
Overall gaze fixation rate	None/mild	617	1	1
	Moderate	72	0.46 (0.13–1.64)	0.15 (0.01–2.25)
	Severe	51	0.75 (0.15–3.81)	4.28 (0.31–58.3)
Gaze fixation rate on the eyes	None/mild	617	1	1
	Moderate	72	1.29 (0.29–5.79)	4.53 (0.30–67.8)
	Severe	51	0.07 (0.008–0.55)^*^	0.02 (0.002–0.38)^**^
Gaze fixation rate on the mouth	None/mild	617	1	1
	Moderate	72	0.63 (0.20–2.03)	2.50 (0.22–28.1)
	Severe	51	1.95 (0.45–8.38)	0.42 (0.05–3.25)

Entered variables: sex, maternal age, and years of education. ASD, autism spectrum disorder; CI, confidence interval. ^**^p < 0.01, ^*^p < 0.05.

We repeated the analysis while replacing the tertiles of ADOS CSS with the tertiles of SA (0–19) and RRB (0–7) domains. No significant associations were observed (Supplementary Table 1).

We also tested the linearity of the association using an ordered logistic regression analysis. No significant association was found (Supplementary Table 2).

### 3.3. Relationship between gaze fixation rate and functioning

**Figure 3:**
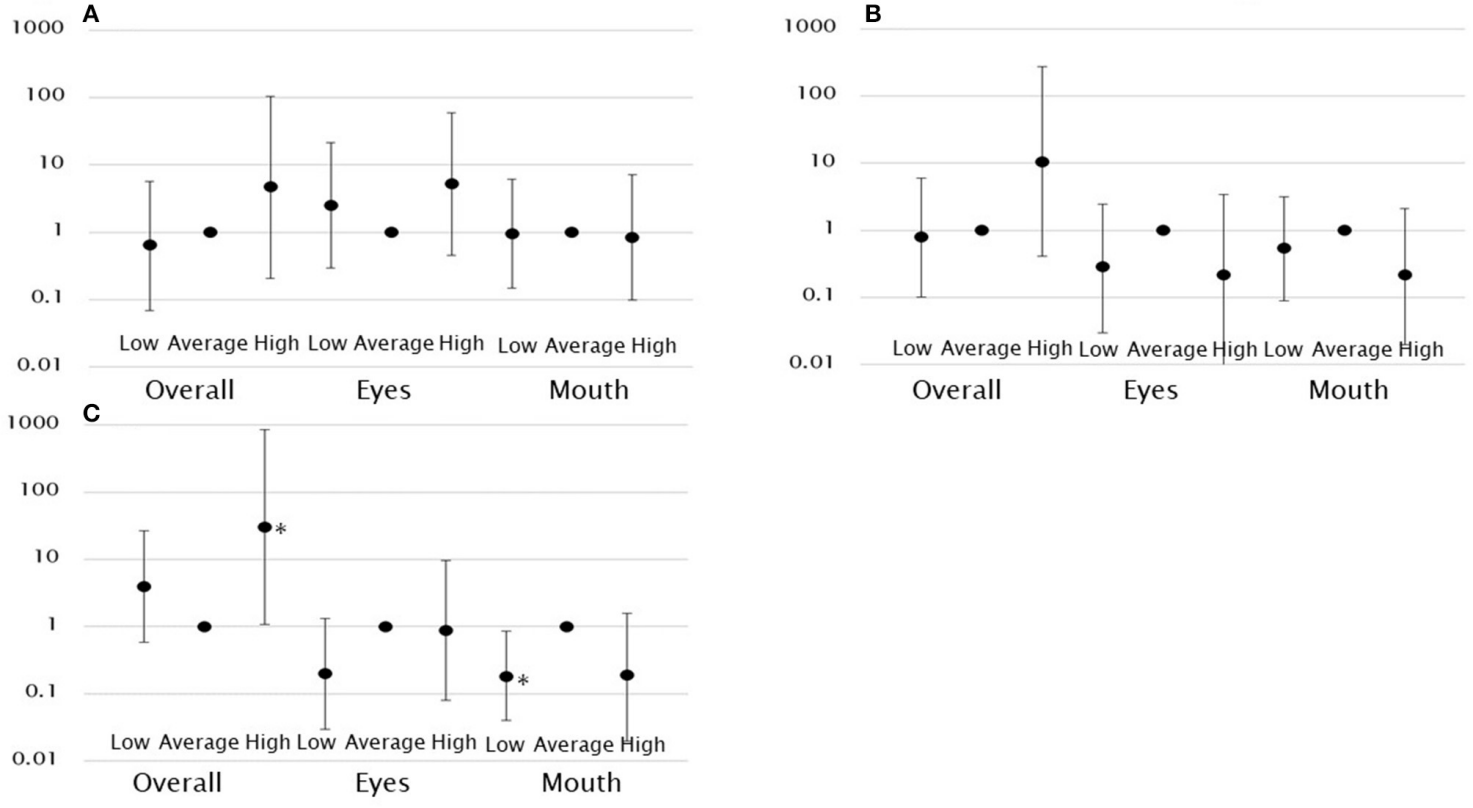
Log-scale representation of odds ratios and 95% confidence intervals indicating the association between gaze fixation rates and functioning classification. There was an association between overall gaze fixation rates and high functioning in socialization and between low gaze fixation rates on the mouth and low functioning in socialization. **(A)** Functioning classification in communication. **(B)** Functioning classification in daily living skills. **(C)** Functioning classification in socialization. *p < 0.05.

A higher gaze fixation rate on the eyes was associated with a significantly higher likelihood of the child being assigned to the high score group in VABS-II communication (model 1: OR, 8.68; 95% CI, 1.98–38.0). However, this relationship was no longer significant after controlling for covariates. No such association was observed in other groups. The gaze fixation rate on the mouth was not associated with the VABS-II communication score (Table 3, Figure 3).

**Table 3:** Association between gaze fixation rate and the functioning score (*n* = 742).

		**N**	**Model 1 Odds ratio (95% CI)**	**Model 2 Odds ratio (95% CI)**
**Communication**				
Overall gaze fixation rate	Low	101	0.86 (0.26–2.82)	0.65 (0.07–5.66)
	Average	537	1	1
	High	104	8.25 (0.98–69.8)	4.72 (0.21–103.6)
Gaze fixation rate on the eyes	Low	101	2.15 (0.58–8.03)	2.52 (0.30–21.4)
	Average	537	1	1
	High	104	8.68 (1.98–38.0)^**^	5.22 (0.46–59.5)
Gaze fixation rate on the mouth	Low	101	0.60 (0.22–1.68)	0.95 (0.15–6.11)
	Average	537	1	1
	High	104	0.80 (0.25–2.60)	0.85 (0.10–7.18)
**Daily living skills**				
Overall gaze fixation rate	Low	91	0.35 (0.12–1.06)	0.79 (0.10–5.96)
	Average	612	1	1
	High	39	2.21 (0.23–21.54)	10.51 (0.41–272.53)
Gaze fixation rate on the eyes	Low	91	0.33 (0.08–1.39)	0.29 (0.03–2.45)
	Average	612	1	1
	High	39	0.99 (0.13–7.40)	0.22 (0.01–3.40)
Gaze fixation rate on the mouth	Low	91	0.62 (0.21–1.77)	0.54 (0.09–3.13)
	Average	612	1	1
	High	39	0.84 (0.18–3.99)	0.22 (0.02–2.12)
**Socialization**				
Overall gaze fixation rate	Low	103	0.74 (0.23–2.33)	3.96 (0.58–27.00)
	Average	584	1	1
	High	55	9.92 (0.85–116.28)	30.44 (1.09–848.48)^*^
Gaze fixation rate on the eyes	Low	103	0.75 (0.20–2.87)	0.20 (0.03–1.34)
	Average	584	1	1
	High	55	5.53 (1.11–27.62)^*^	0.87 (0.08–9.73)
Gaze fixation rate on the mouth	Low	103	0.49 (0.18–1.34)	0.18 (0.04–0.85)^*^
	Average	584	1	1
	High	55	0.62 (0.17–2.28)	0.19 (0.02–1.57)

Entered variables: sex, maternal age, and years of education. CI, confidence interval. ^**^p < 0.01, ^*^p < 0.05.

Neither the gaze fixation rate on the eyes nor the mouth was significantly associated with the VABS-II daily living skill score (Table 3, Figure 3).

A higher gaze fixation rate on the eyes was associated with a significantly higher likelihood of the child being assigned to the high score group in VABS-II socialization (model 1: OR, 5.53; 95% CI, 1.11–27.62). However, this relationship was no longer significant after controlling for covariates. A higher gaze fixation rate on the mouth was associated with a significantly lower likelihood of the child being assigned to the low score group in VABS-II socialization. This was observed after controlling for covariates (model 2: OR, 0.18; 95% CI, 0.04–0.85). This indicates that the lower the gaze fixation rate on the mouth, the more likely the child is to have a functional impairment. In addition, the overall gaze fixation rate was associated with a significantly higher likelihood of the child being assigned to the high score group in VABS-II socialization (model 2: OR, 30.44; 95% CI, 1.09–848.48) (Table 3, Figure 3).

We also tested the linearity of the association using an ordered logistic regression analysis. No significant association was found (Supplementary Table 3).

## 4. Discussion

### 4.1. Summary of the results

The main findings of this study are as follows: in the 6-year-old general population, (1) a low gaze fixation rate on the eyes was predictive of severe ASD symptoms. However, the gaze fixation rate on the mouth was not predictive; and (2) the gaze fixation rate on the eyes was not associated with functioning in the context of communication, daily living skills, or socialization. However, a low gaze fixation rate on the mouth was predictive of poor functioning in socialization. These results were controlled for covariates for the overall gaze fixation rate and rates of gaze fixation on the eyes and on the mouth. The linearity of the association was not supported.

### 4.2. Comparison with previous research regarding the relationship between gaze fixation rate on the eyes and ASD symptoms

Previous studies investigating children with ASD have reported a negative association between the gaze fixation rate on the eyes and ASD symptoms (7, 9, 43–45). This is consistent with the findings of the current study, confirming this association in 6-year-old children from the general population. This is also indicative of the usefulness of eye gaze measurement in non-clinical settings. However, we did not observe any association of gaze fixation rate on the eyes with moderate levels of ASD symptoms, nor did we observe any dose–response relationship. Hence, the low level of gaze fixation rate on the eyes is predictive only of the diagnosable level of ASD symptoms.

Some studies have reported no association of gaze fixation on the eyes with ASD symptoms (8, 46–49). The lack of this association could be ascribed to the stimulus movie clips used in these studies. For instance, these movie clips included distracting backgrounds, including toys and daily life materials, together with the human figure, thus the amount of gaze fixation on the social figures was generally decreased. However, in the current study, we carefully excluded such distractions from our movie clips.

### 4.3. Why was the gaze fixation rate on the mouth and symptoms unrelated?

One study has supported an association between increased gaze fixation on the mouth and severe ASD symptoms (43). However, this has not been substantiated in other studies (44, 46, 49, 50). Other studies have reported an association between decreased gaze fixation on the mouth and severe ASD symptoms (8). Stuart et al. reviewed the hypothesis that children with ASD may inherently avoid gazes directed at them from others (51). Other studies, including ours, have also reported findings regarding the gaze fixation rate on the eyes that are consistent with this hypothesis. However, children participating in these studies are not more likely to direct their gaze to the mouth than to the eye region. Consequently, no associations between the gaze fixation rate on the mouth and ASD symptoms were observed in our studies as well as other relevant studies. Interestingly, gaze fixation on the mouth has been associated with language function and communication ability (22, 52, 53). However, we could not substantiate this finding as we did not observe any association of gaze fixation on the mouth with communicative functioning assessed with VABS-II. Hence, we cannot substantiate the relevance of gaze fixation on the mouth in 6-year-old children to the presence of ASD symptoms or to functioning in communication and daily living skills.

### 4.4. Comparison with previous research regarding the relationship between gaze fixation rate on the mouth and functioning

Two studies have reported an association between gaze fixation on the mouth and functioning in socialization, measured with VABS (8, 46); this finding is consistent with our results. Other studies have failed to replicate this association (49, 50).

The assessment of functioning is significantly influenced by age as the items included in VABS-II are age-specific (25). Hence, the variety of ages of the study participants in the previous studies may also account for the inconsistency of the findings.

More importantly, the inconsistency in study findings can also be ascribed to the presence of various stimuli in the used movie clips. One study reported a positive association, similar to ours. They used a movie clip in which the figure in the clip talks directly to the viewer while looking straight ahead (46). In contrast, another study reporting no association used a movie clip in which two figures talk to each other (49). Important aspects of functioning in socialization, measured with VABS-II, include both the ability to interact with others and the ability to adjust behavior according to the social context (25). Hence, the increased amount of gaze fixation on the mouth may not simply represent social interest or attention. In addition, it may reflect preparedness for the subsequent behaviors provoked by the direct communication of the figure in the movie clip.

### 4.5. Clinical implications

In this study, we observed that the decreased rate of gaze fixation on the eyes predicted severe ASD symptoms, and the decreased rate of gaze fixation on the mouth predicted functional impairment. We can expand this as follows:

ASD symptoms and functional impairment inherently arise from separate bio-psycho-social backgrounds. Hence, we have successfully extracted two physiological endophenotypes, namely, gaze fixation rate on the eyes, which is associated with ASD symptoms, and gaze fixation rate on the mouth, which is associated with functional impairment in socialization. Children with ASD also pay poor attention to both the eyes and mouth (20, 54). When considering poor gaze fixation on the eyes or mouth for clinical prediction, one may take care of the clinical contexts specific to the gaze direction.

### 4.6. Limitations

The first limitation of our study concerns the reliability of the Gazefinder used in this study. The gaze fixation rate used in our study has already been reported in previous studies using Gazefinder as an index that measures the percentage/proportion of time that the subject spends gazing at a specific region (17, 38). Although our results suggest the possibility of diagnosing 6-year-old children with ASD using Gazefinder, its long-term stability requires further investigation.

The second limitation is regarding the research environment. The associations between gaze fixation rate on the eyes or mouth and ASD symptoms and functional impairments might have been influenced by the movie clips used in the study. Children with ASD symptoms might have co-existing sensory idiosyncrasies, and children with functional impairments may have experienced difficulty sitting in front of a screen to continuously watch the clip.

The third limitation pertains to the covariates. In previous studies, when exploring the relationship between gaze fixation rates in children with ASD and ASD symptoms or functional impairments, the nonverbal developmental quotient was utilized as a covariate (46, 52). In this study, targeting the general population, the non-verbal developmental quotient could serve as a covariate for the associations between gaze fixation rates and ASD symptoms or functional impairment, although we had no available data. Therefore, further analyses that consider the characteristics of the participants, including the non-verbal developmental quotient, may be considered.

## 5. Conclusion

This study has elucidated the relevance of a low rate of gaze fixation on the eyes to severe ASD symptoms and of a low rate of gaze fixation on the mouth to functional impairment in socialization in 6-year-old children enrolled from the general population. These findings suggest the possibility of independently assessing preschool children in the general population for clinically relevant endophenotypes predictive of ASD symptoms and functional impairments.

## Data availability statement

The datasets presented in this article are not readily available because the consent given by the participants does not allow publicly delivering/sharing data on an individual level in repositories or journals. Access to the datasets requires approval from HBC Study Executive Committee, Hamamatsu University School of Medicine. Requests to access the datasets should be directed to KJT, kodomo@hama-med.ac.jp.

## Ethics statement

The studies involving humans were approved by the Institutional Review board of Hamamatsu University School of Medicine (20–233). The studies were conducted in accordance with the local legislation and institutional requirements. Written informed consent for participation in this study was provided by the participants' legal guardians/next of kin.

## Author contributions

TM, KJT, TK, and ME conceptualized the idea, designed the study, analyzed and interpreted the data, and drafted the manuscript. KJT, TH, CN, AO, and TN collected the data. TM, KJT, TH, CN, AO, TN, TK, and ME critically revised the manuscript. TK and ME supervised the study. All authors have read and approved the final version of the manuscript.

## In memoriam

We express our deepest gratitude to Professor Emeritus Toshisaburo Nagai (deceased on 9 March 2023) for his support of our research over the years.
