# Supplementary material for: Autism symptoms, functional impairments, and gaze fixation measured using an eye-tracker in 6-year-old children
Source: Front Psychiatry. 2023 Oct 2;14:1250763. doi: 10.3389/fpsyt.2023.1250763 (PMC10577268; doi:10.3389/fpsyt.2023.1250763)
Supplement: Supplementary file 1 [file Table_1.docx]

Supplementary Material

**Autism symptoms, functional impairments, and gaze fixation measured using an eye-tracker in 6-year-old children**

**Toko Mori^*^, Kenji. J. Tsuchiya, Taeko Harada, Chikako Nakayasu, Akemi Okumura, Tomoko Nishimura, Taiichi Katayama, Masayuki Endo**

*** Correspondence:**

Toko Mori

[t-mori@un.shijonawate-gakuen.ac.jp](mailto:t-mori@un.shijonawate-gakuen.ac.jp)

**Supplementary Table 1. Relationship between the severity of the two ASD symptom domains (Social Affect/****Restricted interests, Repetitive Behaviors) and gaze fixation rate (n = 740): Multinomial logistic regression**

|  |  | n | Odds ratio (95% CI) |
| --- | --- | --- | --- |
| Social Affect  Overall gaze fixation rate | Low | 255 | 1 |
|  | Middle | 280 | 0.81 (0.15-4.51) |
|  | High | 205 | 0.25 (0.04-1.71) |
| Gaze fixation rate on the eyes | Low | 255 | 1 |
|  | Middle | 280 | 1.72 (0.34-8.63) |
|  | High | 205 | 1.88 (0.28-12.50) |
| Gaze fixation rate on the mouth | Low | 255 | 1 |
|  | Middle | 280 | 1.13 (0.29-4.46) |
|  | High | 205 | 2.97 (0.58-15.09) |
| Restricted interests, Repetitive Behaviors  Overall gaze fixation rate | Low | 581 | 1 |
|  | Middle | 73 | 2.60 (0.24-27.90) |
|  | High | 86 | 0.22 (0.01-3.37) |
| Gaze fixation rate on the eyes | Low | 581 | 1 |
|  | Middle | 73 | 0.95 (0.11-8.46) |
|  | High | 86 | 6.05 (0.41-88.28) |
| Gaze fixation rate on the mouth | Low | 581 | 1 |
|  | Middle | 73 | 0.35 (0.05-2.25) |
|  | High | 86 | 4.35 (0.39-48.90) |

Social Affect score (tertile): Low=0, Middle=1–4, High=5–19

Restricted interests, Repetitive Behaviors score (tertile): Low=0, Middle=1, High=2–7

Entered variables: sex, maternal age, and years of education.

ASD, autism spectrum disorder; CI, confidence interval.

**Supplementary Table 2. Relationship between the severity of ASD symptoms and gaze fixation rate (n = 740): Ordered logistic regression**

|  | Odds ratio (95% CI) |
| --- | --- |
| Overall gaze fixation rate | 0.94 (0.14-6.26) |
| Gaze fixation rate on the eyes | 0.36 (0.05-2.48) |
| Gaze fixation rate on the mouth | 0.83 (0.16-4.22) |

Entered variables: sex, maternal age, and years of education.

ASD, autism spectrum disorder; CI, confidence interval.

**Supplementary Table 3. Association between gaze fixation rate and the functioning score (n = 742): Ordered logistic regression**

|  | Odds ratio (95% CI) |
| --- | --- |
| Communication  Overall gaze fixation rate | 2.18 (0.40-12.01) |
| Gaze fixation rate on the eyes | 1.30 (0.25-6.83) |
| Gaze fixation rate on the mouth | 0.93 (0.23-3.86) |
| Daily living skills  Overall gaze fixation rate | 3.00 (0.47-19.30) |
| Gaze fixation rate on the eyes | 1.28 (0.20-8.19) |
| Gaze fixation rate on the mouth | 0.84 (0.17-4.15) |
| Socialization  Overall gaze fixation rate | 0.94 (0.17-5.15) |
| Gaze fixation rate on the eyes | 3.59 (0.66-19.47) |
| Gaze fixation rate on the mouth | 1.97 (0.47-8.24) |

Entered variables: sex, maternal age, and years of education.

CI, confidence interval.
